# Granule cells control recovery from classical conditioned fear responses in the zebrafish cerebellum

**DOI:** 10.1038/s41598-017-10794-0

**Published:** 2017-09-19

**Authors:** Koji Matsuda, Masayuki Yoshida, Koichi Kawakami, Masahiko Hibi, Takashi Shimizu

**Affiliations:** 1 Graduate School of Science, Nagoya University, Nagoya, Aichi 464-8602 Japan; 2 Laboratory of Organogenesis and Organ Function, Bioscience and Biotechnology Center, Nagoya University, Nagoya Aichi, 464-8601 Japan; 3 Graduate School of Biosphere Science, Hiroshima University, Higashihiroshima, Hiroshima 739-8528 Japan; 4 Division of Molecular and Developmental Biology, National Institute of Genetics, and Department of Genetics, SOKENDAI (The Graduate University of Advanced Studies), Mishima, Shizuoka 411-8540 Japan

## Abstract

Although previous studies show that the cerebellum is involved in classical fear conditioning, it is not clear which components in the cerebellum control it or how. We addressed this issue using a delayed fear-conditioning paradigm with late-stage zebrafish larvae, with the light extinguishment as the conditioned stimulus (CS) and an electric shock as the unconditioned stimulus (US). The US induced bradycardia in the restrained larvae. After paired-associate conditioning with the CS and US, a substantial population of the larvae displayed CS-evoked bradycardia responses. To investigate the roles of the zebrafish cerebellum in classical fear conditioning, we expressed botulinum toxin or the Ca^2+^ indicator GCaMP7a in cerebellar neurons. The botulinum-toxin-dependent inhibition of granule-cell transmissions in the corpus cerebelli (CCe, the medial lobe) did not suppress the CS-evoked bradycardia response, but rather prolonged the response. We identified cerebellar neurons with elevated CS-evoked activity after the conditioning. The CS-evoked activity of these neurons was progressively upregulated during the conditioning and was downregulated with repetition of the unpaired CS. Some of these neurons were activated immediately upon the CS presentation, whereas others were activated after a delay. Our findings indicate that granule cells control the recovery from conditioned fear responses in zebrafish.

## Introduction

The cerebellum functions as a neuronal learning machine to control various behaviors. Classical conditioning and the role of the cerebellum in learning have been extensively investigated^1, 2^. For instance, the repeated pairing of a conditioned stimulus (CS) (e.g., a tone or light) and an unconditioned stimulus (US) (e.g., an air puff directed into the eyes) leads to CS-evoked eye blinking, and this eyeblink conditioning depends on the cerebellum. During learning, granule cells and Purkinje cells receive two inputs from outside the cerebellum, through mossy fibers (MFs) and climbing fibers (CFs). The MF information is relayed by granule-cell axons, called parallel fibers (PFs), and Purkinje cells integrate the MF/CF information and send the outputs outside the cerebellum through projection neurons (the deep cerebellar nucleus in mammals and eurydendroid cells in teleosts). Repeating the CS and aversive US (e.g., an electric shock) can induce CS-evoked avoidance responses in unrestrained animals (adaptive avoidance learning). However, if animals are restrained, the CS induces freezing behaviors, such as bradycardia. Fear conditioning involves the amygdala in mammals^3, 4^. In zebrafish, the ventral and dorsal habenula are involved in expressing and modifying fear responses^5, 6^. The cerebellum also plays a crucial role in classical fear conditioning. In mammals, lesions of the cerebellar vermis or inferior olive nuclei (IOs), from which the CFs originate, impair the acquisition of conditioned bradycardia responses^7–9^. Inactivating the cerebellar vermis with tetrodotoxin disrupts fear-memory consolidation in rats^10^. Functional imaging in the human brain revealed that the cerebellar midline area is activated when recalling fear episodes^11^. In goldfish, conditioned bradycardia responses are impaired by lesions or by drug-mediated inhibition of the cerebellum^12, 13^. These reports indicate that the cerebellum is involved in classical fear conditioning, including conditioned autonomic regulation, in both mammals and teleosts. However it is not clear which components in the cerebellum control the classical fear-conditioned response, or how they are involved.

The cerebellar neural circuits are generally conserved between zebrafish and mammals^14–16^. Simple cerebellar neural circuits involving granule and Purkinje cells form by 5 days post-fertilization (dpf) in zebrafish early larvae^14, 17^. Electrophysiological studies revealed that Purkinje cells have both simple spikes and complex spikes, representing the MF-PF and CF inputs, respectively, in the early larval stages^18–20^. Consistent with these observations, the cerebellar neural circuitry is activated during adaptation of fictive swimming in the optomotor response paradigm^19^. CF lesions prevent motor adaptation in the early larvae^21^, and activating or inhibiting Purkinje cells affects early larval swimming^22^. These findings imply that the cerebellum controls motor adaptation in the early larval stages. In addition, zebrafish early larvae (6–8 dpf) can acquire classical conditioned responses; associated learning with CS (light) and US (touch) results in a CS-dependent increase in tail movement, and laser ablation of cerebellar neurons blocks this conditioned response^23^. However, zebrafish are also reported to acquire the ability to learn in classical conditioning during the late larval stages^24^. Thus, it is still not clear whether zebrafish in the early larval stages are capable of classical fear conditioning.

We previously established zebrafish transgenic (Tg) lines that express modified Gal4 in the cerebellar neural circuits^25^. Here we investigated the roles of the larval zebrafish cerebellum in classical fear conditioning using the granule-cell-specific Gal4 line.

## Results

### Late-stage larval zebrafish exhibited classical fear-conditioned responses

**Figure 1:**
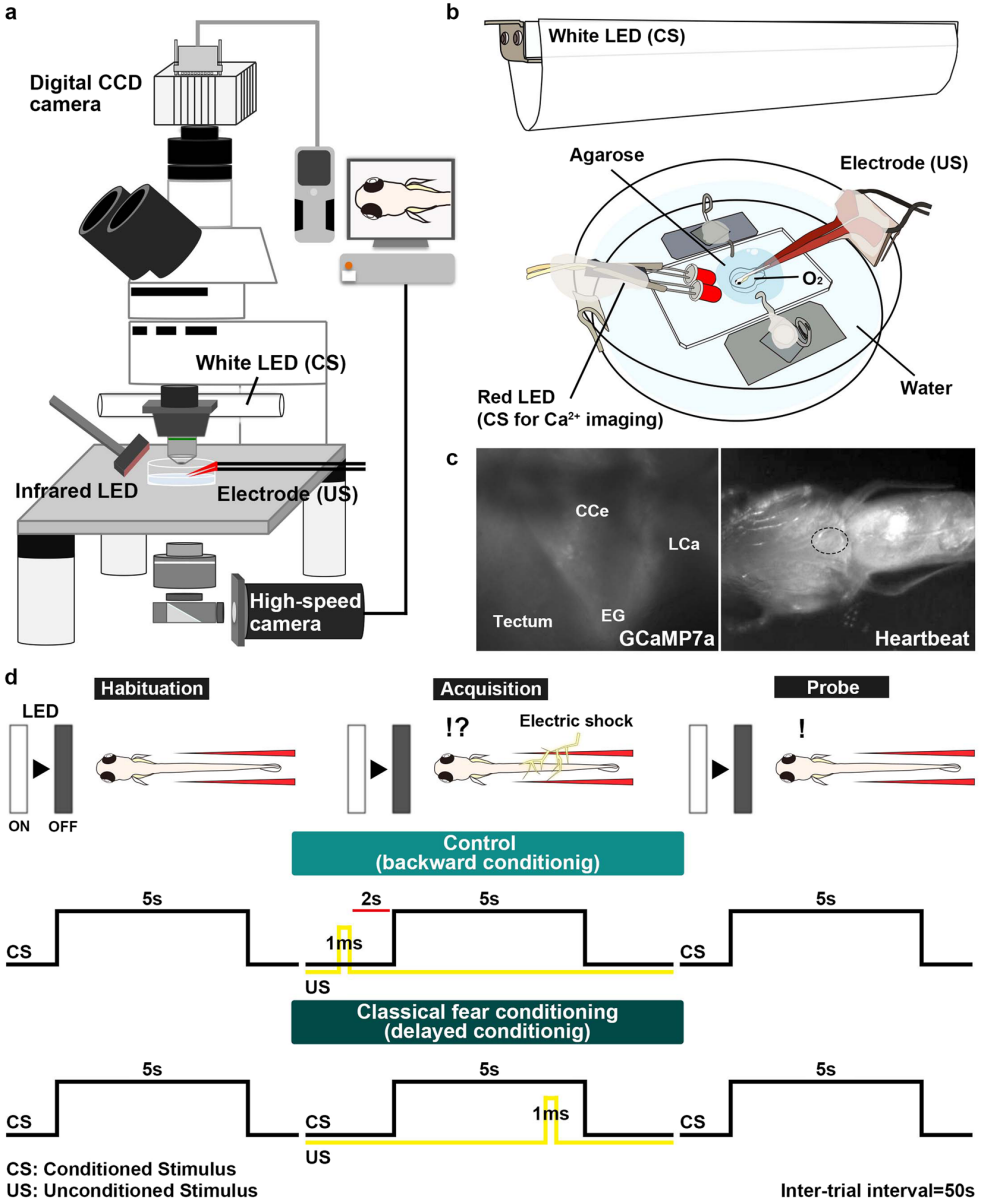
Method for investigating classical fear conditioning in zebrafish. (**a**,**b**) Schematic of the experimental setup for classical fear conditioning using zebrafish larvae at about 20 dpf. The larva was restrained in agarose gel, and O_2_ gas was provided from below to support respiration. Extinguishment of a white or red LED was given as the CS, and an electric shock was given as the US. (**c**) Imaging of Ca^2+^ signals of cerebellar neurons (left panel); and heartbeats (HB, right panel) in *Tg(elavl3:GAL4-VP16); Tg(UAS:GCaMP7a)* larvae. Images showing the movement of the heart were obtained from underneath with an infrared digital camera. The heart is indicated by a dotted circle. CCe, corpus cerebelli; EG, eminentia granularis; LCa, lobus caudalis cerebelli. (**d**) Experimental paradigm for delayed classical fear conditioning. In the habituation session, CS alone was given for 5 s per trial; 10–15 trials were performed. In the acquisition session, a paired CS and US (1-ms electric shock given 4 s after the CS onset) was given in each trial; 20 trials were performed. In the probe session, the CS alone was given in each trial and 10 trials were performed. As a control (backward conditioning), the US was given 2 s before the CS onset in the acquisition session. Trials were separated by a 50-s interval. A 50-s interval was also provided between sessions.

**Figure 2:**
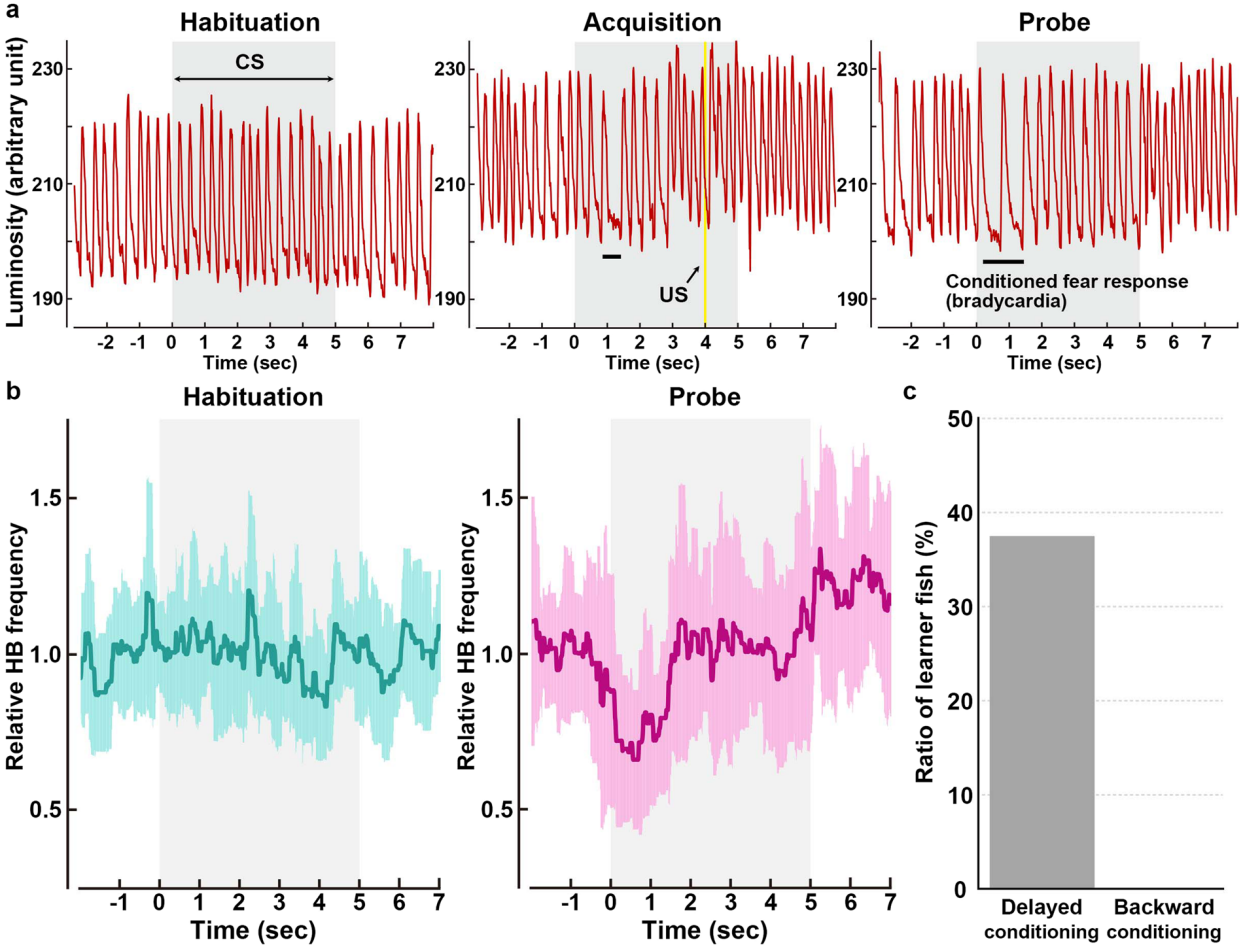
Late-stage zebrafish larvae acquired the conditioned fear response. (**a**) A typical HB pattern in the classical fear conditioning, showing the heart movement in the 15^th^ trial of the habituation session, the 16^th^ trial of the acquisition session, and the 8^th^ trial of the probe session, and the timing of the CS (gray boxes) and US (yellow line). About 20-dpf wild-type larvae were used. Representative data obtained from a single individual larva are shown. The x-axis shows the time (sec). The y-axis shows the heartbeats as monitored by luminosity (arbitrary unit) at an appropriate region of the heart in IR video. Each peak represents one heartbeat. In the acquisition trial, the US elicited the bradycardic response. Note that in the late acquisition session (middle panel) and in the probe session (right panel), bradycardia, denoted by horizontal bars, occurred after the start of the CS presentation. (**b**) The relative HB frequency in the habituation and probe sessions. The HB frequencies from 10 trials (6^th^–15^th^ trials of habituation; 1^st^–10^th^ trials of the probe session) were averaged, divided by the control HB frequency (the average HB frequency for 2 s before the CS), and indicated as a relative HB frequency in the graph (y-axis). The relative HB frequency was determined every 11.1 ms. The shaded error region shows the standard deviation (SD); gray boxes show the timing of the CS. Representative data obtained from a single individual larva are shown. We compared the relative HB frequency between the habituation and probe sessions, and identified larva with a significantly reduced relative HB frequency during the first 2 s of the CS period as learners. (**c**) Percentage of wild-type fish showing CS-dependent bradycardia (learners) in the delayed fear conditioning and the control backward conditioning. 15 of 40 larvae were learners in the delayed conditioning, and none of 10 were learners in the backward conditioning.

The repeated pairing of a CS and an aversive US leads to bradycardia and other escape behaviors in response to the CS in teleosts and mammals. To understand the neural circuits involved in fear conditioning, we used a delayed fear-conditioning paradigm with zebrafish larvae (Fig. 1) since the delayed fear conditioning paradigms were commonly used to study the role of cerebellum in learning for animals including goldfish^10, 12, 13^. The extinguishment of a white LED light was used as the CS, and an electric shock was used as the US (Fig. 1d). Classical fear conditioning consists of three sessions: habituation, acquisition, and probe. In the habituation session, the larvae were exposed to a 5-s CS for 10–15 trials. In the acquisition session, the CS was paired with the US (1 ms, delivered 4 s after the CS onset) for 20 trials. In the probe session, the larvae were exposed to the CS alone for 10 trials. In a control experiment (backward conditioning), the US was delivered 2 s before the CS. A 50-s interval was provided between trials or sessions. We monitored the larva’s heartbeat (HB) to assess the conditioned responses (Supplementary Video S1). Preliminary experiments indicated that early-stage larvae showed hardly any conditioned bradycardic responses (*n* = 0/10 for 5–9 dpf; *n* = 3/12 for 10–16 dpf). It is difficult to study juvenile and adult fish, which are one month old or older, since oxygen should be provided continuously for sufficient branchial respiration. Thus, we decided to study the behavior of late-stage larvae, which were 17–25 days old (we refer to them hereafter as about 20-dpf larvae). About 20-dpf larvae that showed typical paired-associate learning exhibited arousal bradycardia response to the first CS in the habituation session. The arousal response disappeared after 1–2 trials, and the HB was unaffected by the CS thereafter (Fig. 2a, left panel). At the beginning of the acquisition stage, bradycardia responses were elicited only by the US. After around 10^th^–15^th^ trial in the acquisition session, the conditioned bradycardia occurred shortly after the CS onset and the HB recovered during the CS presentation (Fig. 2a, middle panel). The conditioned response persisted in the probe session where no US was applied (Fig. 2a, right panel). We quantified and compared the HB frequency between the habituation and probe sessions (Fig. 2b). We defined “learners” as zebrafish larvae whose HB frequency was unchanged in response to the CS during the 6^th^–15^th^ trials of the habituation session but was significantly reduced (bradycardia response) during the 1^st^–10^th^ trials of the probe session (Welch’s *t*-test, *P* < 0.05). The results showed that 37.5% (*n* = 15/40) of around 20-dpf wild-type zebrafish larvae were learners. The conditioned response on the HB frequency did not decrease or fade in the 6^th^–10^th^ trials, compared to the 1^st^–5^th^ trials, in the probe session (Supplementary Fig. S1). No learners were observed when the larvae were subjected to the backward conditioning (*n* = 0/10, Fig. 2c, Supplementary Fig. S2).

### Granule cells are involved in classical fear conditioning

**Figure 3:**
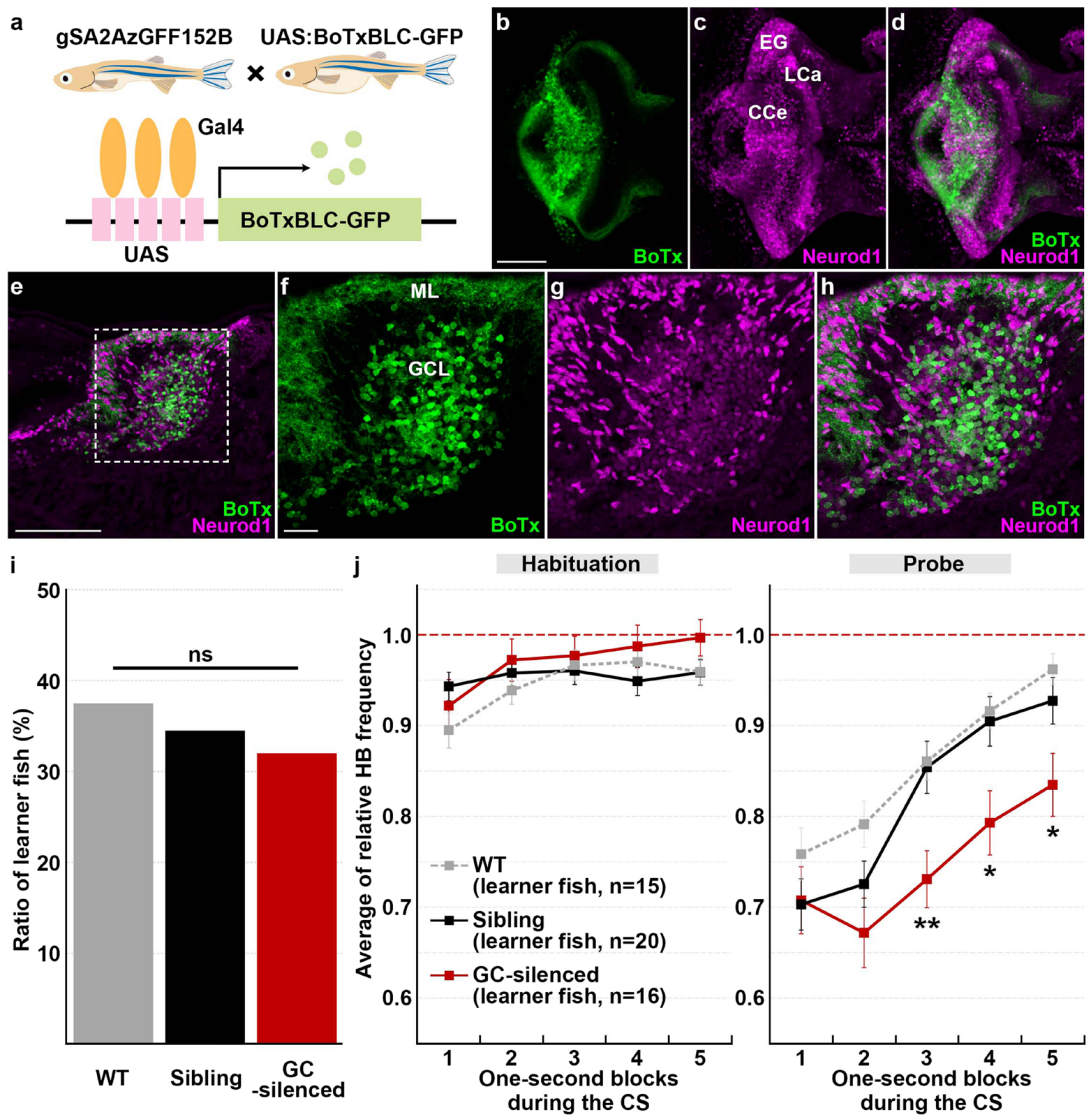
Granule-cell inhibition prolonged conditioned fear responses. (**a**) Schematic of granule-cell inhibition. Tg larvae with inhibited granule-cell activity (GC-silenced larvae) were obtained by crossing the granule-cell-specific Gal4 line gSA2AzGFF152B and the *Tg(UAS:BoTxBLC-GFP)* line, which expresses a fusion protein of GFP and the light chain of botulinum toxin light B (BoTxBLC-GFP) in a Gal4-dependent manner. (**b**–**h**) BoTxBLC-GFP expression in 20-dpf GC-silenced larvae. Immunostaining of the brain (whole-mount, **b**–**d**) and sagittal sections (**e**–**h**) with anti-GFP (green) and Neurod1 (magenta) antibodies, showing the cerebellar regions. Dorsal views with anterior to the left. (**f**–**h**) Higher magnification views of the dotted box in (**e**). Neurod1 signals mark granule-cell nuclei. About a half of the mature granule cells in the CCe (located in the granule-cell layer) expressed BoTxBLC-GFP in the GC-silenced larvae (Supplementary Fig. S3, Table S1). Scale bars: 100 μm in (**b**) (applied to **c**,**d**); 100 μm in (**e**); 20 μm in (**f**) (applied to **g**,**h**). GCL, granule-cell layer; ML, molecular layer (see Fig. 1 for other abbreviations). (**i**) Percentage of fish showing CS-dependent bradycardia (learner fish); 15 of the 40 wild-type (WT) larvae were learners, 16 of the 50 GC-silenced larvae were learners, and 20 of the 58 sibling GFP-negative larvae were learners. There was no significant difference in the learner rate among wild-type, the GC-silenced and the sibling groups (*P* = 0.8615, Fisher’s exact test). ns represents no significance. (**j**) CS-evoked bradycardia responses in wild-type, the GC-silenced, and their sibling learner fish. Relative HB frequency during 1 s period of the 5 s CS presentation (average of each one-second block) is shown. Average of the data from 10 trials in the habituation and probe sessions was calculated and plotted in graphs. The graphs show the average and standard errors (SE) of the data from wild-type larvae (*n* = 15, gray dotted), the GC-silenced larvae (*n* = 16, red), and their sibling larvae (*n* = 20, black). The CS-evoked bradycardia responses during the probe session differed between the GC-silenced and the sibling learner groups (** represents *P* < 0.01 and * represents *P* < 0.05, two-way repeated measures ANOVA with Bonferroni’s post-hoc test). Both the GC-silenced and sibling larvae exhibited bradycardia in response to the CS during the probe session. The bradycardic response of the GC-silenced larvae was prolonged in the probe sessions. Note that the change of relative HB frequency in wild-type learner fish during the habituation and probe sessions was similar to that in the sibling learner fish.

We investigated the cerebellum’s role in classical fear conditioning by inhibiting granule-cell function. To this end, we crossed the granule-cell-specific Gal4 line gSA2AzGFF152B^25^ and the *Tg(UAS:BoTxBLC-GFP*) line, which expresses a fusion protein of GFP and the light chain of botulinum toxin B (BoTxBLC-GFP); the botulinum toxin inhibits synaptic release in a Gal4-dependent manner^26^ (Fig. 3a). We selected larvae expressing BoTxBLC-GFP in the granule cells (GC-silenced larvae) at 5 dpf, and reared the GC-silenced larvae and their siblings together in the same tank until about 20 dpf. We confirmed that BoTxBLC-GFP was expressed only in the cerebellum of the larval brain (Fig. 3b–d, Supplementary Fig. S3). About a half of the granule cells which are marked by Neurod1 and located in the granule cell layer (GCL) in the CCe of the GC-silenced larvae expressed BoTxBLC-GFP, whereas only a minor population (about 1.5%) of the granule cells in the caudo-lateral lobes (eminentia granularis [EG] and lobus caudalis cerebelli [LCa]) expressed BoTxBLC-GFP (Fig. 3b–h, Supplementary Fig. S3, Table S1). There was no apparent difference in swimming behavior between the GC-silenced larvae and their siblings (Supplementary Fig. S4).

When subjected to the classical fear conditioning, a similar proportion of GC-silenced and their sibling larvae showed CS-evoked bradycardia during the probe session (Fig. 3i,j). There was no significant difference in learning rates among wild-type, the GC-silenced, and their sibling larvae (*P* = 0.8615, Fisher’s exact test, Fig. 3i). There was no difference in HB frequency during the CS presentation in the habituation session between the GC-silenced and their sibling larvae (Fig. 3j). However, the CS-evoked bradycardia responses during the probe session differed significantly between the two groups; while the HB recovered quickly after the CS-evoked bradycardia response in the sibling larvae, it remained significantly lower in the GC-silenced learners during CS presentation (two-way repeated measures ANOVA: group effect *P* = 0.0463, group x time interaction *P* = 0.00769; Fig. 3j, Supplementary Fig. S1, Supplementary Video S2). These data indicate that inhibiting granule-cell transmission in the CCe impaired the recovery from bradycardic responses, and suggest that granule cells play a role in recovery from the conditioned bradycardia response.

### Ca^2+^ imaging of the zebrafish cerebellum during classical fear conditioning

**Figure 4:**
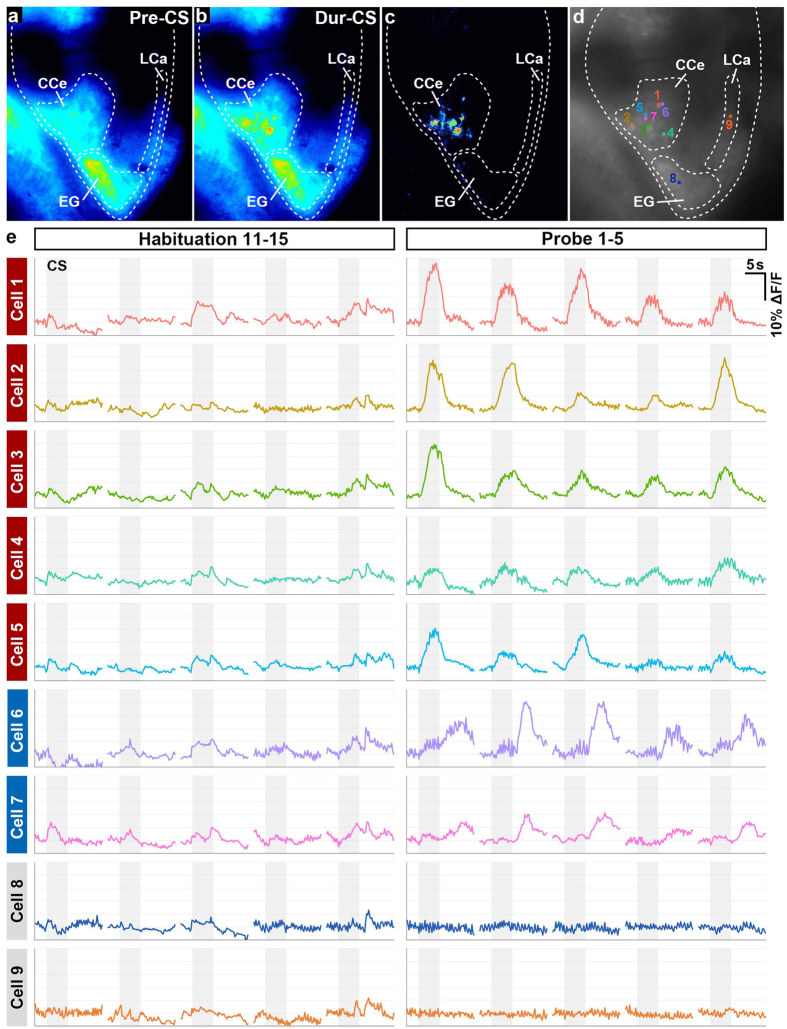
Cerebellar neurons were activated during classical fear conditioning. (**a**,**b**) Ca^2+^ imaging. GCaMP7a fluorescence intensity in *Tg(elavl3:GAL4-VP16); Tg(UAS:GCaMP7a)* larva before (pre-CS, **a**) and during (dur-CS, **b**) the CS in the probe sessions. The images were taken 2 s before and 2.5 s after the start of the CS presentation. Average images from nine trials in the probe session are shown. (**c**) The “dur-CS minus pre-CS” image generated by subtraction. Note that the CS evoked upregulated fluorescence intensity only in the CCe. (**d**,**e**) Time course of cerebellar neuronal activity. The fluorescence intensity from nine cerebellar neurons in a single larva was monitored during the habituation (11^th^–15^th^ trials) and probe (1^st^–5^th^ trials) sessions. Graph shows the ΔF/F, which was calculated by dividing the change in fluorescence intensity at the indicated time point by the average intensity at 2 s before the CS (**e**). Gray boxes indicate the timing of the CS presentation. The position of the neurons is shown in (**d**). During the probe session, the CS evoked upregulated fluorescence in seven neurons (Cells 1–7) in the CCe. There was no change in fluorescence between the habituation and probe sessions in a neuron in the EG (Cell 8) or in one in the LCa (Cell 9). (See Fig. 1 for abbreviations).

To monitor neuronal activity in the zebrafish cerebellum during classical fear conditioning, we crossed the pan-neuronal Gal4-driver *Tg(elavl3:GAL4-VP16)* line^27^ and a reporter *Tg(UAS:GCaMP7a)* line^28^. We analyzed about 20-dpf Tg larvae. To monitor GCaMP7a fluorescence, the larval brain is usually illuminated with a blue excitation light with a spectrum that overlaps the white LED we used for the CS. To avoid this overlap, we used a red LED instead of the white LED, and extinguishment of the red LED as the CS in the cued fear conditioning. We found that the paired-associated learning with the red LED also induced the CS-evoked bradycardia responses (*n* = 3/10). The change in GCaMP7a fluorescence intensity (ΔF/F) was calculated to estimate the neuronal activity (Fig. 4a–d). In a typical case, we detected spontaneous activity of the cerebellar neurons during the habituation session, but did not detect any upregulation of the ΔF/F in response to the CS. In the probe session, however, the CS evoked an increase in ΔF/F (more than 3% on average during the CS presentation across the first five trails) in some cerebellar neurons, which were termed “conditioning-associated neurons” (Supplementary Video S3). A substantial population of the larvae that were subjected to the conditioning displayed more than four conditioning-associated neurons (*n* = 9/25). Examples of the conditioning-associated neurons are shown in Fig. 4d,e (Cells 1–7). These neurons were located only in the CCe, and not in the caudo-lateral lobes (EG and LCa) (Fig. 4c–e). We found no conditioning-associated neurons in the control backward conditioning.

### The temporal regulation of conditioning-associated neurons

**Figure 5:**
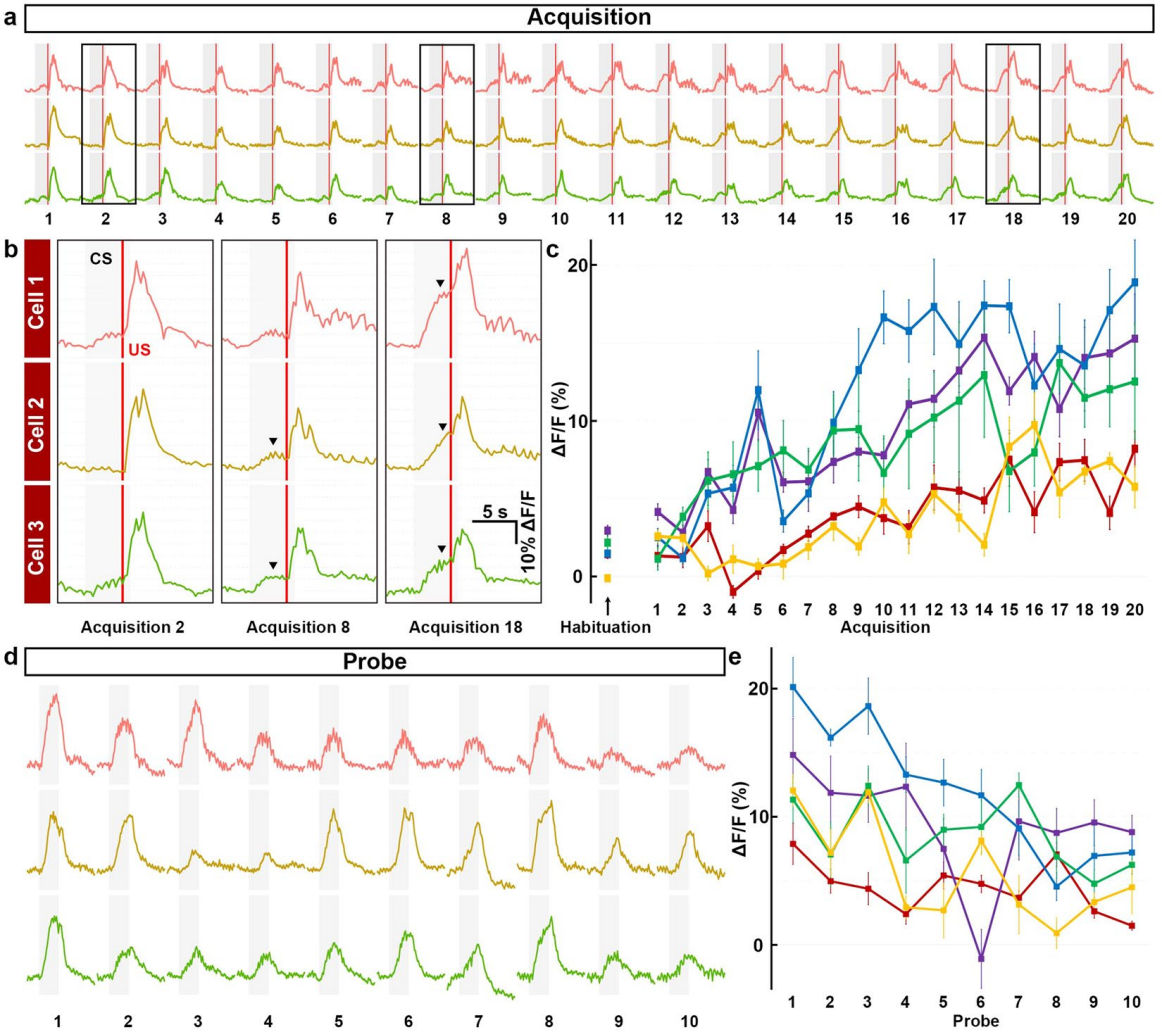
Time course of the activity of conditioning-associated neurons during conditioning. Cells that displayed an increase in the neuronal activity (ΔF/F of CaMP7a, more than 3% on average during CS presentation, across the 1^st^–5^th^ trials) in the probe session were termed “conditioning-associated neurons”. (**a**) Neuronal activity (ΔF/F) during the acquisition (1^st^–20^th^ trials) session, showing data from three conditioning-associated neurons. Gray boxes and red lines indicate the CS presentation periods and the timing of the US, respectively. (**b**) High-magnification views of the boxes in (**a**) (the 2^nd^, 8^th^, and 18^th^ trials in the acquisition session). CS-evoked neural activities (arrowheads) gradually increased during the acquisition session. Note that the fluorescence was strongly upregulated upon the US. (**c**) The activity (ΔF/F) of the conditioning-associated neurons gradually increased during the acquisition session. Five conditioning-associated neurons were selected from each larva. Graph shows the average and SE of the ΔF/F of these five neurons during the first 4 s of the CS for each trial (ΔF/F_CS0-4_), calculated for five individual larvae (indicated by different colors). (**d**) Activity (ΔF/F) of the conditioning-associated neurons during the probe session, showing data from three conditioning-associated neurons. Gray boxes indicate the timing of the CS presentation. (**e**) The activity of the conditioning-associated neurons decreased during the probe session. Graph shows the average ΔF/F_CS0-4_ from five conditioning-associated neurons from each of five larvae (indicated by different colors).

We next investigated the activity of the conditioning-associated neurons over time (Fig. 5). After Ca^2+^ imaging, we identified the conditioning-associated neurons, which showed CS-evoked activity (ΔF/F) during the probe session. We then retrospectively identified these neurons and examined their activity during the acquisition session. The data from three of the conditioning-associated neurons are shown in Fig. 5a,b. In the 2^nd^ trial in the acquisition session, although the US evoked an increase in the ΔF/F, there was little or no increase in response to the CS. In the 8^th^ trial, we detected a slight CS-evoked increase in ΔF/F (in two of the three neurons). In the 18^th^ trial, the CS-evoked ΔF/F was markedly increased in all three neurons. To examine whether this type of regulation occurred in other conditioning-associated neurons, we analyzed data from the five larvae that had conditioning-associated neurons, and calculated the average ΔF/F of five conditioning-associated neurons from each larva. Since the US upregulated the ΔF/F in most cerebellar neurons (Fig. 5a,b), we used the ΔF/F value during the first 4 s of the CS (before the US) to measure the CS-evoked response. The CS-evoked ΔF/F upregulation in the conditioning-associated neurons gradually increased during the acquisition session in all five larvae (Fig. 5c). We also examined the ΔF/F during the 1^st^–10^th^ trials of the probe session (Fig. 5d,e), and found that the CS-evoked activity of the conditioning-associated neurons gradually decreased during the probe session (Fig. 5e). These data suggest that memory (conditioning-associated activity) forms progressively during paired-associate conditioning and gradually disappears with the repeated presentation of the unpaired CS.

Simultaneous monitoring of the ΔF/F activity and the HBs revealed that four of the nine larvae that had more than four conditioning-associated neurons showed the bradycardia responses at multiple trials in the probe session. In these larvae, the bradycardia responses occurred when conditioning-associated neurons appeared in the acquisition and probe sessions (a typical example is shown in Supplementary Figs S5, S6 and S7). The larvae with no conditioning-associated neurons showed no conditioned bradycardia responses.

### Two types of conditioning-associated neurons

**Figure 6:**
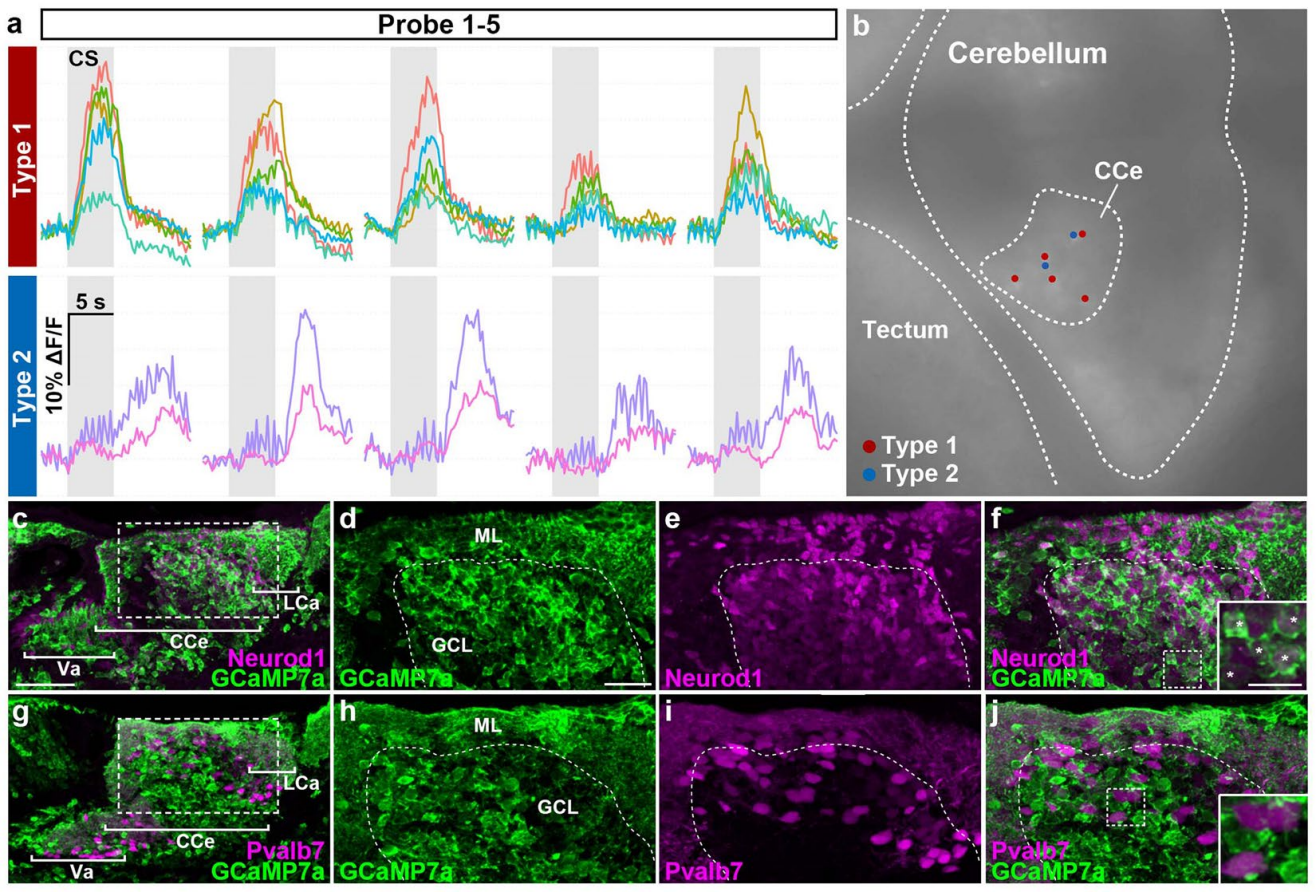
Two types of conditioning-associated neurons. We found two types of conditioning-associated neurons that were activated by the CS. Type I neurons responded quickly to the CS, while type II neurons were activated after a delay. (**a**) Neuronal activity (ΔF/F) of type I and II neurons of *Tg(elavl3:GAL4-VP16); Tg(UAS:GCaMP7a)* larvae during the probe session (1^st^–5^th^ trials), showing data from five type I neurons and two type II neurons. Gray boxes indicate the timing of the CS presentation. (**b**) Location of the type I (red dots) and II neurons (blue dots) in the CCe. These neurons were located close to each other in the CCe. (**c**–**f**) Immunostaining of *Tg(elavl3:GAL4-VP16); Tg(UAS:GCaMP7a)* about 20-dpf larval brains with anti-GFP (green) and anti-Neurod1 (magenta) antibodies. (**d**–**f**) Higher magnification views of the dotted box in (**c**). The inset in (**f**) is a higher magnification view of the dotted box in (**f**). Neurod1 signals mark granule-cell nuclei. Note that *Tg(elavl3:GAL4-VP16); Tg(UAS:GCaMP7a)* fish expressed GCaMP7a in most of the granule cells in the Va/CCe/LCa (Va: valvula cerebelli) (white asterisks in the inset in **f**). (**g**–**j**) Immunostaining of *Tg(elavl3:GAL4-VP16); Tg(UAS:GCaMP7a)* brains with anti-GFP (green) and anti-Pvalb7 (magenta) antibodies. (**h**–**j**) Higher magnification views of the dotted box in (**g**). The inset in (**j**) is a higher magnification view of the dotted box in (**j**). Pvalb7 signals mark both the neurites (axons and dendrites) and somata of Purkinje cells. No Purkinje cells expressed GCaMP7a (Table S2). Pvalb7^+^ cells in the GCL are also Purkinje cells as migration of Purkinje cells was not completed at the stage. See Figs 1 and 3 for abbreviations. Scale bars: 50 μm in (**c**) (applied to **g**); 20 μm in (**d**) (applied to **d**–**f**, **h**–**j**); 10 μm in the inset in (**f**) (applied to the inset in **j**).

We found two types of conditioning-associated neurons in the cerebellum. Type I neurons responded immediately to the CS presentation, while type II neurons were activated in a delayed fashion (Fig. 6a). The activity (ΔF/F) of type I neurons began increasing upon presentation of the CS, peaked during the late CS phase, and decreased soon after the CS ended. The activity of type II neurons increased slightly upon the CS presentation but increased strongly after the CS ended. Type I and II neurons were located close to each other in the CCe (Fig. 6b). We next investigated the identity of these conditioning-associated neurons by comparing the expression of GCaMP7a and markers for granule and Purkinje cells (Neurod1 and parvalbumin7: Pvalb7) in the Tg larvae (Fig. 6c–j). Most of the elavl3:GCaMP7a-positive somata co-expressed Neurod1 but not Pvalb7, which marks neurites and somata of Purkinje cells (Fig. 6f,j, and Supplementary Table S2). Considering the location of the conditioning-associated neurons in the CCe, our data imply that the conditioning-associated neurons we observed in the Tg line were granule cells. This identification is consistent with the idea that the *elavl3* (also known as HuC) promoter is active in immature and newly generated neurons in teleosts^29^, and that granule cells are continuously generated in the larvae^17, 30^. Our data suggest that there are two types of conditioning-associated granule cells, which can be called early and late responders.

## Discussion

### Classical fear conditioning in zebrafish

In this study, we found that a high percentage of late-stage zebrafish larvae (around 20 dpf) acquired classical fear-conditioned responses (37.5%, *n* = 15/40), whereas few early larvae acquired this conditioning (*n* = 0/10 for 5–9 dpf; *n* = 3/12 for 10–16 dpf). Early larvae were reported to efficiently acquire a conditioned response using LED lighting for the CS and body touch for the US^23^. Other studies using zebrafish at different stages revealed that fear conditioning was first reliably achieved around 3 weeks post-fertilization^24^. Since the learning conditions were different, it is difficult to determine when robust fear conditioning generally occurs. In this report, we concluded that robust conditioning with an electric shock as US can occur only in late larval stages, as published already^24^. The conditioned bradycardia response observed in mammals and goldfish depends on the cerebellar neural circuits, suggesting that the conditioned bradycardia response is a cerebellum-mediated fear response that is conserved among vertebrates. By 5 dpf in zebrafish, simple cerebellar neural circuits form^14^, and Purkinje cells show simple and complex spikes, reflecting inputs from granule cells and CFs^18–20^. These observations indicate that the cerebellar neural circuits are functional at this early stage, but raise the question of why early larvae do not learn efficiently. The CFs in early-stage larvae encode simple information such as visual and motor signals but do not encode signals to adapt a motor behavior such as retinal slip^19^. It should take time for the cerebellar circuits to mature enough to control complex behaviors such as fear conditioning.

### The cerebellum is involved in classical fear conditioning

Inhibiting granule-cell activity in the CCe did not interfere with fear conditioning, but rather prolonged the CS-evoked bradycardia response (Fig. 3). Consistent with this observation, we identified conditioning-associated neurons in the CCe that were activated by the CS during the probe session; these neurons were likely to be granule cells (Figs 4 and 6, Supplementary Table S2). Although the botulinum toxin was expressed in about a half of the mature granule cells in the CCe in GC-silenced Tg larvae (Fig. 3f–h, Supplementary Fig. S3, Table S1), our data indicated that at least a portion of the granule cells in the CCe were involved in controlling the recovery of conditioned response. The onset of CS-evoked bradycardia was relatively normal in the GC-silenced larvae, but their recovery from the bradycardia was delayed (Fig. 3j), suggesting that granule cells control the recovery process. This finding is in contrast to previous reports that inhibiting activity in the cerebellum abolishes the conditioned bradycardia response in goldfish^12, 13, 31^ and mammals^7, 8, 10^, and that inhibiting the IOs or the interpositus nucleus (IN, a deep cerebellar nucleus) in mammals abrogates fear conditioning^9, 10^, which all suggest a positive role of the cerebellar neural circuits in conditioned fear responses. The discrepancy between these studies and our findings might be caused by the difference in the cell populations inhibited; the previous inhibition experiments perturbed cerebellar function non-selectively. The cerebellar neural circuits include complex components such as inhibitory Purkinje cells, excitatory granule cells, and various types of synaptic plasticity, making it difficult to dissect the effects of non-selective inhibition of the cerebellar neural circuits on classical fear conditioning. In the present study, we selectively inhibited granule-cell-mediated synaptic transmission (Fig. 3), and revealed a previously unrecognized role of granule cells in regulating recovery from the conditioned bradycardia response. The continuous inhibition of the granule-cell transmission from an early larval stage (Fig. 3) might cause a rewiring of the cerebellar neural circuits resulting in alleviation of the phenotypes. Nevertheless, our data indicate that granule cells are involved in the recovery of the conditioned response even if the defective circuits are compensated.

### Functional domains in the zebrafish cerebellum

Previous studies that inhibited the cerebellar vermis in mammals^7, 8, 10^ suggest that the vermis is responsible for the autonomic response in classical fear conditioning. We found that granule cells in the CCe play a role in the conditioned bradycardia response in zebrafish (Fig. 3). In these animals, caudal Purkinje cells are reported to regulate the optokinetic response whereas the rostro-medial Purkinje cells regulate swimming behavior, suggesting functional domains within the zebrafish cerebellum^22^. Granule cells in the CCe send axons toward rostro-medial Purkinje cells, granule cells in the caudo-lateral lobes project toward caudal Purkinje cells in the CCe and crest cells in the dorsal hindbrain^32, 33^, and caudo-lateral Purkinje cells extend axons toward the vestibular system^14, 22, 25, 34^, indicating that the cerebellar neural circuits differ between the rostro-medial and caudo-lateral cerebellum. Our findings suggest that the rostro-medial cerebellar circuit (which involves the granule cells in the CCe) in the teleost cerebellum is functionally equivalent to the vermis in the mammalian cerebellum, and that it controls conditioned autonomic responses.

### How do cerebellar neurons learn?

Although we found that the US activated cerebellar neurons in both the CCe and EG/LCa, the CS did not activate cerebellar neurons at the beginning of the acquisition session (Fig. 5). These results imply that few if any cerebellar neurons respond to the unpaired CS, although we might have overlooked a low level of CS-evoked activation. Learning processes occur at various synaptic levels in the cerebellar neural circuits. Long-term depression (LTD) and long-term potentiation (LTP) in the PF-Purkinje cell synapses, LTP in the MF-IN synapses, and LTP in the MF-granule cell synapses are thought to play important roles in cerebellar learning^35–40^. Our findings suggest that the learning process, which implies the integration of information from the CS and US, occurs at or upstream of the level of inputs to the granule cells. This mechanism may cooperate with the LTD/LTP at other synaptic levels to control the conditioned fear response. When the unpaired CS was repeated in the probe session, the activity of conditioning-associated neurons was downregulated (Fig. 5e). Granule-cell activation may also be downregulated at the same synaptic level as the integration that occurs during learning. Although the conditioning-associated neurons progressively acquired CS-evoked activity during the acquisition session (Fig. 5), the larvae demonstrated the bradycardia response in the middle of the acquisition session (Fig. 2). The simultaneous monitoring of the neuronal activity and the HBs revealed that in all the cases, the conditioned responses appeared when the conditioning-associated neurons were present (Supplementary Figs S6 and S7). Our observations imply that there is a threshold for activating cerebellar granule cells (conditioning-associated neurons). When the activity of the learner granule cells exceeds this threshold, zebrafish begin exhibiting the conditioned bradycardia responses. They continue the conditioned responses until the activity of the learner granule cells becomes below the threshold (it did not occur within the 10 trials of the probe session, Supplementary Fig. S1).

### How do granule cells control the conditioned bradycardia response?

The LTP in PF–Purkinje cell synapses is reported to increase during fear conditioning in mammals^38, 41^. However, the suppression of simple spikes of Purkinje-cell activity during eyeblink conditioning in mammals is also reported^42, 43^. Although fear conditioning in goldfish increases the simple spikes in some Purkinje cells, more of these cells show a reduction in simple spikes^44^, suggesting that in teleosts, Purkinje-cell activity is mostly suppressed during fear conditioning. The reduced Purkinje-cell activity probably upregulates the activity of the projection neurons, which may be responsible for expressing the conditioned fear responses. Our data suggest that granule cells are involved in the recovery from the conditioned bradycardia response (i.e., in suppressing the bradycardia response). This raises the intriguing possibility that the increased activity of conditioning-associated granule cells activates Purkinje cells, subsequently suppressing the eurydendroid cells and conditioned responses. This possibility should be clarified by experiments using functional imaging and the manipulation of Purkinje and eurydendroid cell activities during fear conditioning.

We identified two types of conditioning-associated neurons involved in fear conditioning (Fig. 6). Given that these neurons are involved in the recovery from the conditioned bradycardia response, type I and type II neurons may play roles in the early and late stages of the recovery, respectively. Type I neurons may encode a recovery program that is coordinately initiated with the program that induces the bradycardia, while type II neurons may play a role in a feedback mechanism in which the sensory information or the command for bradycardia secondarily activates a recovery program. Another possibility is that type I and II neurons share the same MF inputs, as these neurons are located close to each other (Fig. 6). The different sensitivities of type I and II neurons to MF inputs may be attributed to the distinct time course of the activation of these neurons. Selective stimulation or inhibition of type I or II neurons should reveal the functions of these neurons in fear conditioning.

Our findings indicate that zebrafish provide a good model for investigating the role of cerebellar neural circuits in classical fear conditioning. Future studies using transgenic and mutant zebrafish to dissect the functions of each component of the cerebellar circuits should contribute to a better understanding of the emotional functions of the cerebellum.

## Methods

### Ethics statement

The animal experiments in this study were approved by the Nagoya University Animal Experiment Committee and were conducted in accordance with the Regulations on Animal Experiments from Nagoya University.

### Zebrafish

Wild-type zebrafish (*Danio rerio*) with the Oregon AB genetic background and the previously reported Tg lines gSA2AzGFF152B^25^, *Tg(elavl3:GAL4-VP16)* ^*nns6*^ ^27^, *Tg(UAS:GCaMP7a)* ^*zf415*^ ^28^, and *Tg(UAS:BoTxBLC-GFP)* ^*icm21*^ ^26^ were used. We used zebrafish larvae at about 20 dpf. For *in vivo* Ca^2+^ imaging, Tg fish on the *casper* (*mitfa* ^*w2*^ *; roy* ^*a9*^) background were used. The zebrafish were maintained in environmentally controlled rooms at the Bioscience and Biotechnology Center, Nagoya University on a 14–10-h light-dark cycle (light 9 am to 11 pm; dark 11 pm to 9 am). All experiments were conducted during the light phase of the cycle.

### Classical fear conditioning

For fear conditioning, the zebrafish larva was kept in a 1000-ml tank at the regular breeding temperature (27 °C) for over an hour and then anesthetized in 0.02% tricaine methanesulfonate. The larva was then embedded in 4% agarose (low gelling temperature Type VII-A, Sigma-Aldrich) with 1/10 Evans solution (134 mM NaCl, 2.9 mM KCl, 2.1 mM CaCl_2_, 1.2 mM MgCl_2_, and 10 mM Hepes pH 7.8) in a 90-mm-diameter petri dish on the bottom of which an acrylic sheet (1.0-mm-thick) with a hole was placed (Fig. 1a,b). Electrodes (0.5-mm-thick tapered stainless-steel plates) were placed lateral to the larval tail. 0.1 ml of 99% oxygen gas was injected through a syringe with 27 gauge needle into the space between the agarose and the petri dish bottom once at the beginning of the experiment to support respiration. The larva was covered with breeding water and kept on the stage of an upright microscope (Olympus BX51; Olympus, Japan), with an objective lens (XL Fluor 2×/340; numerical aperture [NA] 0.14, Olympus) and a high-speed infrared CCD camera (GZL-CL-41C6M-C, Point Grey, Canada) underneath the stage, for 1 h at 27 °C. An infrared light-emitting diode (LED, LDL2-33 × 8IR850; CCS Inc., Japan) was placed above the stage to illuminate the larva, and infrared movie images of the heart region were acquired at 90 frames per second (fps) with StreamPix6 software (Norpix, Canada). White or red LEDs (peak 640 nm; Kingbright, Taiwan) powered by a DC 8.8–9.0 V power supply (E3631A; Agilent Technologies, USA) were placed to illuminate the fish. Extinguishment of the white LED light was presented as the CS. The electric shock US was a 1-ms rectangular pulse at 80 V/cm applied via the stimulation electrodes with a combination of pulse generator (MASTER-9; A.M.P.I., Israel) and stimulus isolator (ISO-Flex; A.M.P.I.). The timing of the CS and US was controlled with a DAQ interface (USB-6008; National Instruments Co., USA) and laboratory-made software written in LabVIEW (National Instruments Co.). The US was not lethal or damaging for the larvae since they swam normally when freed from the agarose and developed normally after finishing the conditioning experiments.

### Photocardiography

The HB of the larva was monitored from underneath using the infrared LED and the CCD camera (Fig. 1a,c *)*. The infrared movie images were converted to serial JPEG images by StreamPix6. The appropriate images were selected visually and analyzed with a program in LabVIEW. A landmark was fixed on the periphery of the heart with the LabVIEW program, and the variation of the luminosity on the landmark was converted to numerical data that were loaded to the LabChart software (AD Instruments). Each peak in the data (Fig. 2a) represents one heartbeat; the top and bottom of the peaks represent diastolic and systolic phases of the cardiac cycle, respectively. The HB frequency was calculated by the LabChart software. Normal HB frequencies (the average HB frequency for 2 s before the CS in the habituation session) in the wild type, GC-silenced, their sibling, and *Tg(elavl3:GAL4-VP16); Tg(UAS:GCaMP7a)* larvae were 3.37 ± 0.54 Hz (mean ± SD, *n* = 15), 2.89 ± 0.61 Hz (*n* = 16), 2.92 ± 0.56 Hz (*n* = 20), 3.23 ± 1.12 Hz (*n* = 5), respectively. No significant differences in the HB frequencies among the groups were observed (*P* = 0.1194, one-way ANOVA). To minimize the individual variability, a ratio of HB frequency against the average HB frequency for 2 s before the CS was calculated and shown as a relative HB frequency in Figs 2 and 3.

### Ca^2+^ imaging

The cerebellum of a *Tg(elavl3:GAL4-VP16); Tg(UAS:GCaMP7a)* larva embedded in 4% agarose was observed with a water-immersion objective lens (UMPlanFL 20 × W, NA 0.50, Olympus) equipped with a cooled CCD camera (ORCA-R2, Hamamatsu Photonics, Japan). An Olympus 130 W U-HGLGPS light-guide-coupled illumination system was used for the emission light. One hour was allowed for adaptation, and the larva was illuminated with blue light for 5 min before the conditioning session began for habituation of the excitation light. Fluorescence images with 1344 × 1023 px were acquired in a single focal plane at 4 fps with an exposure time of 250 ms using StreamPix6 (Fig. 1a,c). The images were analyzed by a laboratory-made program in LabVIEW as follows. Around five neurons that increased in the fluorescence intensity were usually identified in each larva by visual inspection of fluorescence images. Regions of interest (ROIs) were manually set for these neurons, and the fluorescence intensities in the ROIs were converted to numerical data by the LabVIEW program. Fluorescence intensities were determined for the cells at the same position during the habituation and acquisition sessions. If there was movement in the brain during conditioning, images of the brain were aligned using ImageJ (http://imagej.nih.gov/ij/) with the TurboReg plugin. Changes in fluorescence intensities were expressed as ΔF/F, which was calculated by dividing the fluorescence intensity at the indicated time point by the average intensity measured 2 s before the CS. Typical responder cells displayed CS-evoked increase in ΔF/F up to 20%. Cells that displayed more than 3% (average) increase in the ΔF/F during the CS presentation across the 1^st^–5^th^ trials in the probe session were defined as conditioning-associated neurons.

### Swimming performance test

About 20-dpf larvae were transferred into a weighing dish (100 mm × 70 mm × 13 mm) containing 10 ml water. Underneath the weighing dish, white LEDs were arrayed for the light source. After habituation for 10 min, swimming zebrafish larvae were recorded by a video camera (15 fps) for one minute. The head position of each larva was tracked using the video analysis and modeling tool, Tracker (http://physlets.org/tracker/). Distance and direction of the head movements between consecutive two frames were calculated by Microsoft Excel and the R software package (3.2.3) (https://www.r-project.org/). An event showing more than 90 degree in the direction change of consecutive two movements was counted as a turning. Average swimming speed and turning frequencies were calculated by Microsoft Excel and the R software package.

### Immunostaining

The following antibodies were used for immunostaining: anti-GFP (1:1000, rat, Nacalai Tesque, Japan, Cat# 04404-84) or anti-GFP (1:1000, rabbit, MBL international, Cat# 598) for BoTxBLC-GFP, anti-GFP (1:1000, rabbit, MBL international) for GCaMP7a, anti-Neurod1 (1:400, mouse, ascites)^17^, and anti-parvalbumin 7 (1:1000, mouse monoclonal, ascites)^14^. Larvae and cryostat sections were immunostained as described previously^14, 17^. The following secondary antibodies were used: Alexa Fluor 488 goat anti-rat (H + L, Molecular Probes, Thermo Fisher Scientific, USA, Cat#A11006), CF488A anti-rabbit (H + L, Biotium Inc., USA, Cat#20019), and Alexa Fluor 568 goat anti-mouse IgG (H + L, Molecular Probes, Thermo Fisher Scientific, USA, Cat#A11031). Some fixed samples were optically cleared with SeeDB reagent as previously reported^45, 46^.

### Statistics

Welch’s *t*-test, Fisher’s exact test, one-way ANOVA, and two-way repeated measures ANOVA with Bonferroni’s post-hoc test were performed using the R software package.

## Electronic supplementary material

Supplementary Information

Video S1

Video S2

Video S3
