# Supplementary material for: Granule cells control recovery from classical conditioned fear responses in the zebrafish cerebellum
Source: Sci Rep. 2017 Sep 19;7:11865. doi: 10.1038/s41598-017-10794-0 (PMC5605521; doi:10.1038/s41598-017-10794-0)
Supplement: Supplementary file 1 — Supplementary Information [file 41598_2017_10794_MOESM1_ESM.pdf]

Supplementary Information

"Granule cells control recovery from classical conditioned fear responses in the zebrafish cerebellum"

Koji Matsuda[1,2], Masayuki Yoshida[3], Koichi Kawakami[4], Masahiko Hibi[1,2,*], & Takashi Shimizu[1,2]

[1]Graduate School of Science, Nagoya University, Nagoya, Aichi 464-8602, Japan
[2]Laboratory of Organogenesis and Organ Function, Bioscience and Biotechnology Center, Nagoya University, Nagoya Aichi 464-8601, Japan
[3]Graduate School of Biosphere Science, Hiroshima University, Higashihiroshima, Hiroshima 739-8528, Japan
[4]Division of Molecular and Developmental Biology, National Institute of Genetics, and Department of Genetics, SOKENDAI (The Graduate University of Advanced Studies), Mishima, Shizuoka 411-8540, Japan

*Corresponding author: Masahiko Hibi, Bioscience and Biotechnology Center, Nagoya University, Furo, Chikusa, Nagoya, Aichi 464-8601, Japan, Tel: +81-52-789-5198, Fax: +81-52-789-5053, E-mail: hibi@bio.nagoya-u.ac.jp

Supplementary Tables

Table S1 Expression of BoTxBLC-GFP in the cerebellum of gSA2AzGFF152B; *Tg(UAS:BoTxBLC-GFP)* larvae.

	Region	BoTxBLC-GFP^{+} cells	Neurod1^{+} cells	Ratio (%)
Sample 1	CCe	561	1112	50.45
	LCa	0	184	0
	EG	10	446	2.24
Sample 2	CCe	327	768	42.58
	LCa	0	145	0
	EG	4	316	1.27
Sample 3	CCe	671	1289	52.06
	LCa	3	206	1.46
	EG	7	547	1.28

Sagittal sections (14 μm) of the brain from three 20-dpf gSA2AzGFF152B; *Tg(UAS:BoTxBLC-GFP)* larvae were immunostained with anti-GFP and anti-Neurod1 (a granule cell marker) antibodies. From each larval brain, two lateral and three medial sections that correspond to the EG and the CCe/LCa region were selected (Supplementary Fig. S3d), and the number of BoTxBLC-GFP^{+} or Neurod1^{+} cells in the EG, CCe, or LCa was counted (in the CCe, the number of the cells in the GCL was counted). The ratio of BoTxBLC-GFP^{+} cells to Neurod1^{+} cells is indicated.

Table S2 Expression of GCaMP7a in the cerebellum of *Tg(elavl3:GAL4-VP16); Tg(UAS:GCaMP7a)* larvae.

	**GCaMP7a$^+$ cells**	**GCaMP7a$^+$Neurod1$^+$ cells**	**Ratio (%)**
Sample 1	305	281	92.13
Sample 2	311	276	88.75
Sample 3	293	269	91.81

	**GCaMP7a$^+$ cells**	**GCaMP7a$^+$ Pvalb7$^+$ cells**	**Ratio (%)**
Sample 1	295	0	0
Sample 2	288	0	0
Sample 3	306	0	0

Sagittal sections (14 μm) of the brain from three 20-dpf *Tg(elavl3:GAL4-VP16); Tg(UAS:GCaMP7a)* larvae were immnostained with anti-GFP (for GCaMP7a), and anti-Neurod1 (upper table, Fig. 6c-f) or Pvalb7 antibodies (a Purkinje cell marker, lower table Fig. 6g-j). Five typical sections were selected from each brain, and the number of GCaMP7a$^+$ cells, GCaMP7a$^+$ Neurod1$^+$ cells, and GCaMP7a$^+$ Pvalb7$^+$ cells was counted. The ratio of GCaMP7a$^+$ Neurod1$^+$ cells to GCaMP7a$^+$ cells, or GCaMP7a$^+$ Pvalb7a$^+$ cells to GCaMP7a$^+$ cells is indicated. Note that about 90 % of the GCaMP7a$^+$ cells co-expressed Neurod1 and no Purkinje cells expressed GCaMP7a.

Supplementary Figures

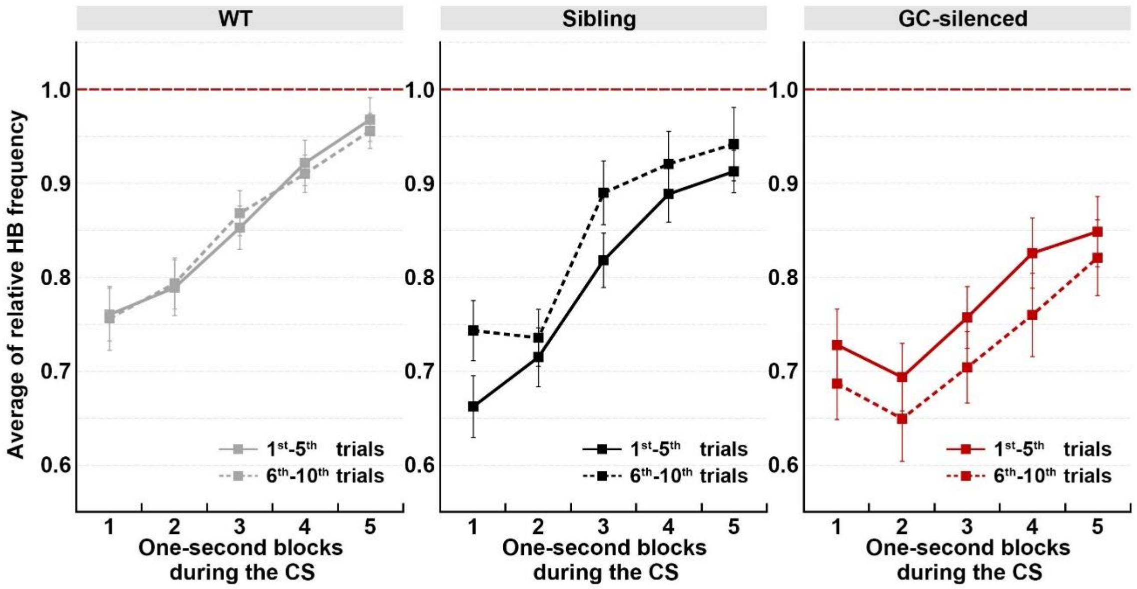

Figure S1 The conditioned response was stable during the 10 trials in the probe session. Relative HB frequency in the 1st-5th trials and 6th-10th in the probe sessions. The graphs show the average and standard errors (SE) of the data from wild-type (WT, n = 15, gray), the GC-silenced [gSA2AzGFF152B; *Tg(UAS:BoTxBLC-GFP)*] (n = 16, red), and their sibling larvae (n = 20, black). The conditioned response on the relative HB frequency did not significantly decrease or fade in the 6th-10th trials (dotted lines), compared to 1st-5th trials (solid lines) (wild-type: group effect P = 0.952; sibling: group effect P = 0.193; GC-silenced: group effect P = 0.325, two-way repeated measures ANOVA).

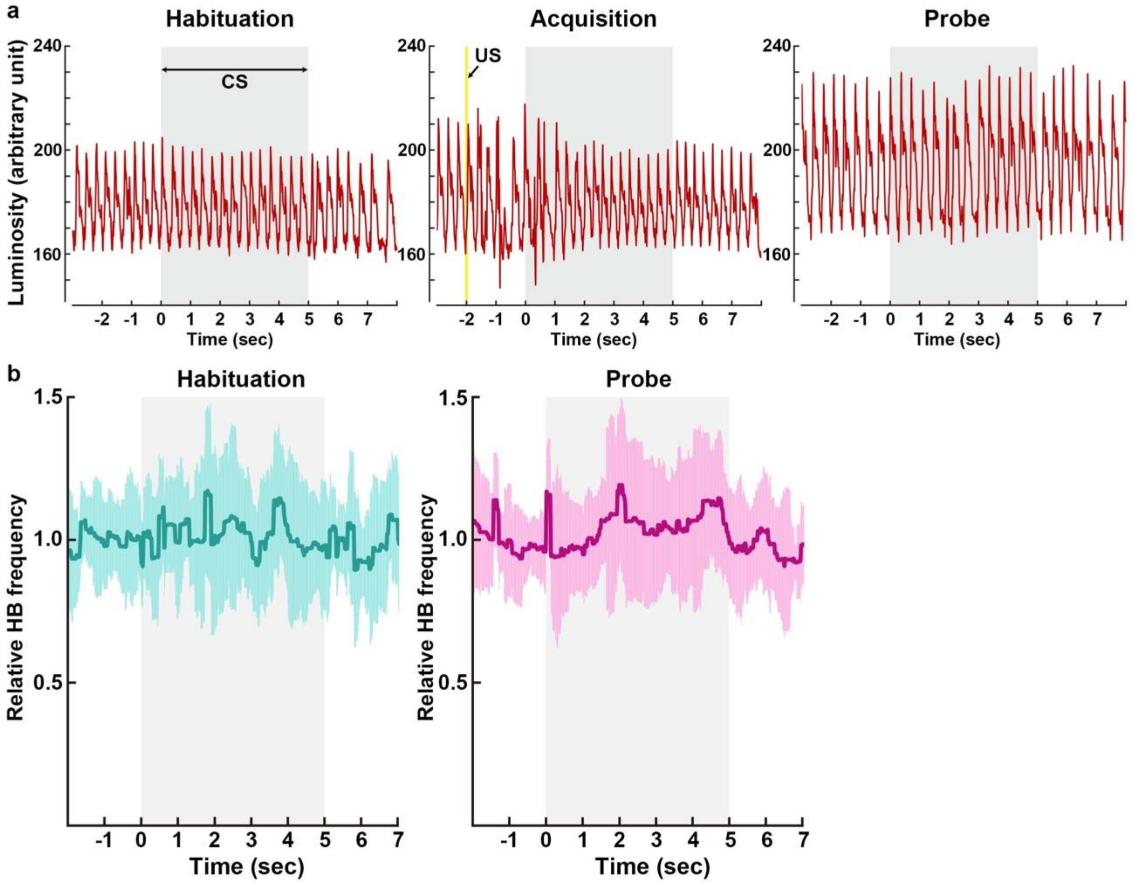

Figure S2 Late-stage zebrafish larvae did not acquire the conditioned fear responses in the backward conditioning.

(**a**) A typical HB pattern in the backward conditioning, showing the heart movement in each trial of the habituation, acquisition, and probe sessions and the timing of the CS (gray boxes) and US (yellow line). About 20-dpf wild-type larvae were used. Representative data obtained from a single individual larva are shown. The x-axis shows the time (sec). The y-axis shows the heartbeats as monitored by luminosity (arbitrary unit) at an appropriate region of the heart in IR video. Each peak represents one heartbeat. Note that in the probe session, bradycardia did not occur after the start of the CS presentation.

(**b**) The relative HB frequency in the habituation and probe sessions. The HB frequencies from 10 trials (6th-15th trials of habituation; 1st-10th trials of the probe session) were averaged, divided by the control HB frequency (the average HB frequency for 2 s before the CS), and indicated as a relative HB frequency in the graph (y-axis). The relative HB frequency was determined every 11.1 ms. The shaded error region shows the standard deviation (SD); gray boxes show the timing of the CS. Representative data obtained from a single individual larva are shown.

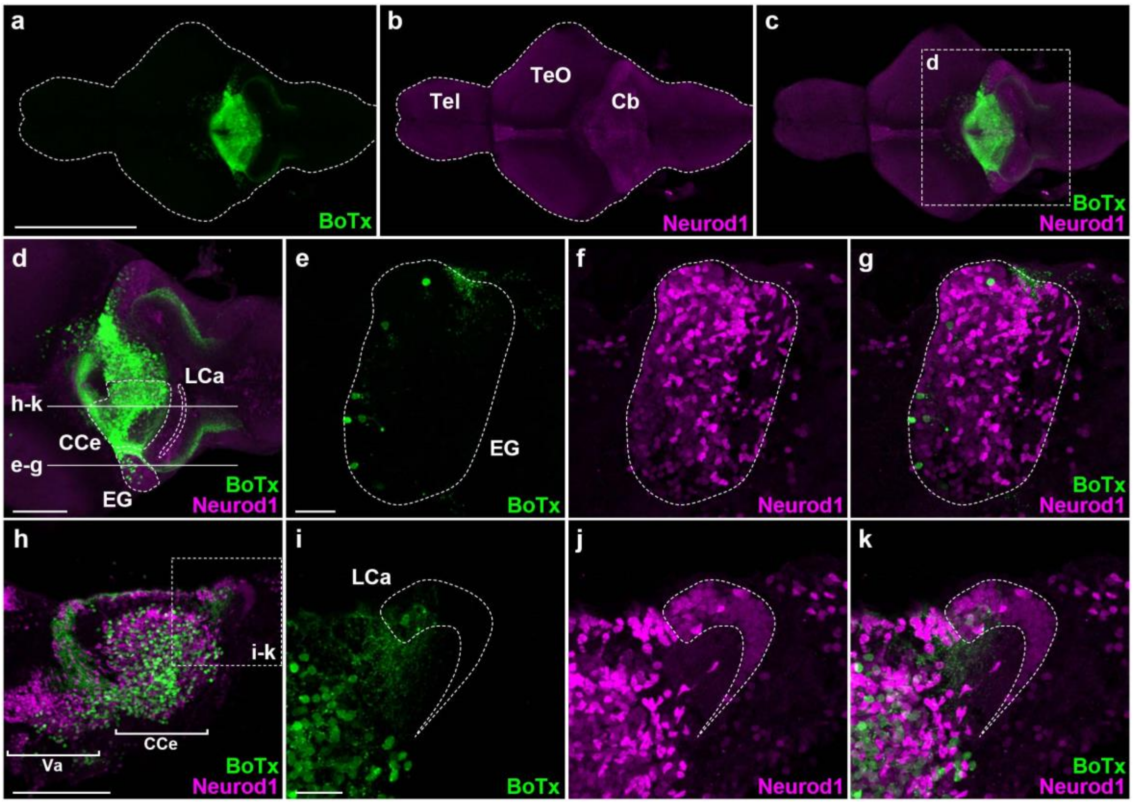

Figure S3 Expression of BoTxBLC-GFP in the about 20-dpf GC-silenced larvae was largely confined to the granule cells in the cerebellum.

(**a-c**) Immunostaining of the whole brain with anti-GFP (green) and Neurod1 (magenta) antibodies. Neurod1 signals mark granule-cell nuclei. Note that the BoTxBLC-GFP expression are detected only in the cerebellum. Dorsal views with anterior to the left. Tel, telencephalon; TeO, optic tectum; Cb, cerebellum. (**d**) Higher magnification views of the dotted box in (**c**). (**e-g**) Sagittal sections at the levels shown in (**d**). Only a small number of Neurod1^{+} cells expressed BoTxBLC-GFP in the EG. (**h-k**) Sagittal sections at the levels shown in (**d**). (**i-k**) Higher magnification views of the dotted box in (**h**). Only a few Neurod1^{+} cells expressed BoTxBLC-GFP in the LCa. CCe, corpus cerebelli; EG, eminentia granularis; LCa, lobus caudalis cerebelli. Scale bars: 400 μm in (**a**) (applied to **b**, **c**); 100 μm in (**d**); 20 μm in (**e**) (applied to **f**, **g**); 100 μm in (**h**); 20 μm in (**i**) (applied to **j**, **k**). See Fig. 1, 3 for abbreviations.

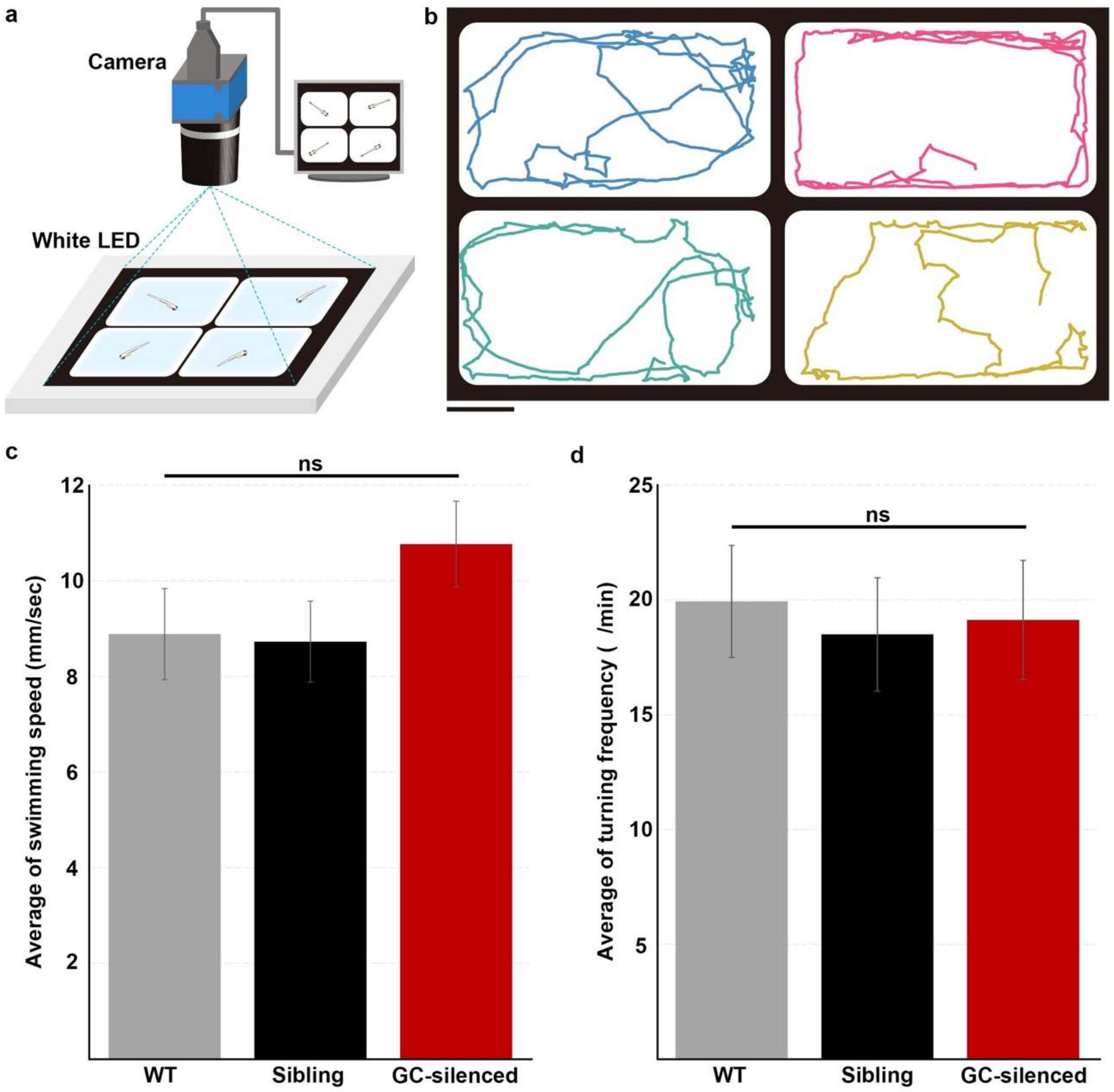

Figure S4 Swimming behavior was not affected in the GC-silenced larvae.
(**a**) Schematic of the experimental setup for swimming performance test using zebrafish larvae at about 20 dpf. The head position of each larva was recorded by a video camera (15 fps) and its trajectories were analyzed. (**b**) Trajectory of wild-type larvae for one min during free swimming. Wild-type (WT), the GC-silenced, and their sibling larvae were examined (*n* = 16 each group). Trajectory of four wild-type larvae is shown. Scale bars: 20 mm. Distance and direction of the head position between consecutive two frames was calculated. (**c**) Average of swimming speed. There was no significant difference between the three groups (*P* = 0.2162, one-way ANOVA). (**d**) Average of turning frequency. An event showing more than 90 degree change in the direction change was counted as a turning. There was no significant difference between the three groups (*P* = 0.9207, one-way ANOVA). ns represents not significant.

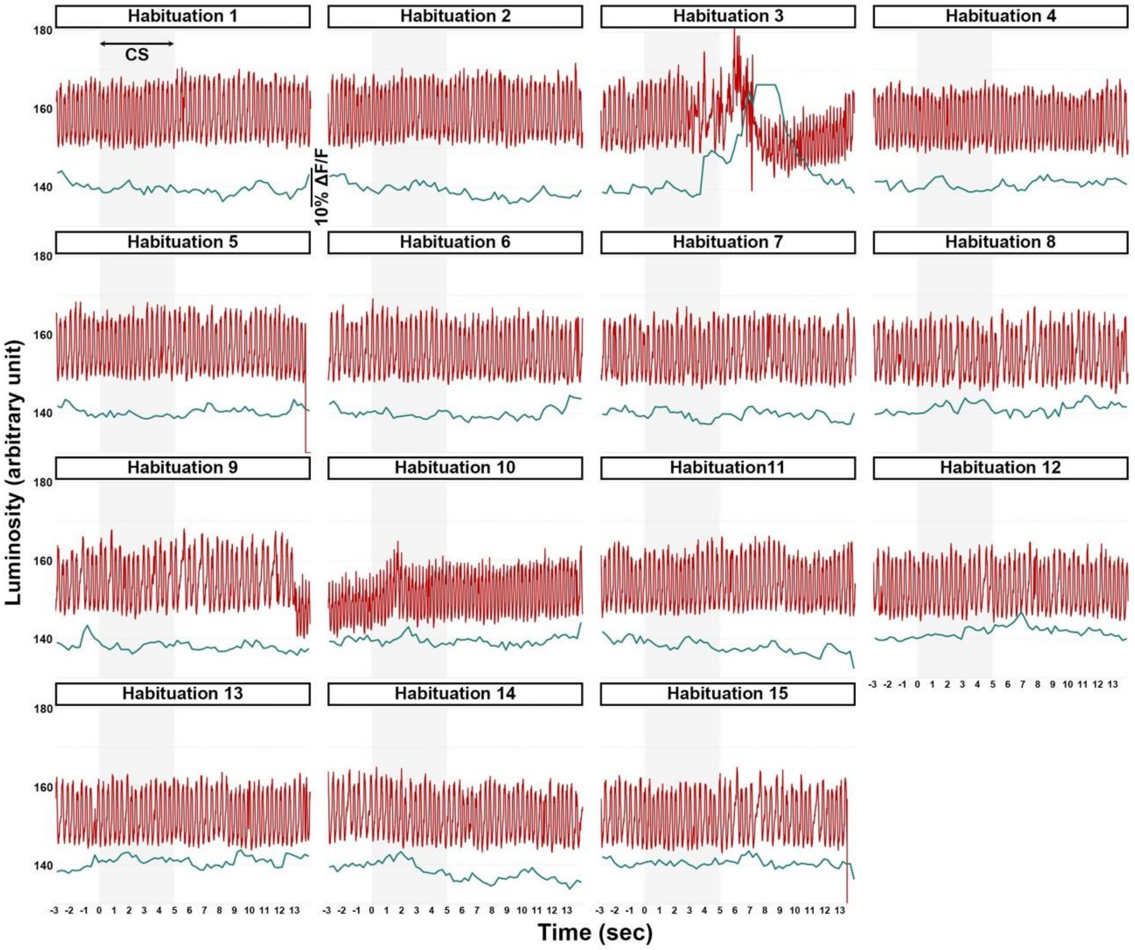

Figure S5 Simultaneous monitoring of the neuronal activity and the HB pattern of a learner larva in the habituation session.

The HB pattern and the fluorescence intensity of GCaMP7a of 25 *Tg(elavl3:Gal4-VP16); Tg(UAS:GCaMP7a)* larvae were simultaneously monitored. One larva was identified as a learner. The HB pattern (red lines) and the ΔF/F activity of a type I conditioning-associated neuron (green lines) in the learner in the habituation session are shown. Those in the acquisition and probe sessions are shown in Supplementary Fig. S6 and S7, respectively. The x-axis shows the time (sec). The y-axis shows the HBs as monitored by luminosity (arbitrary unit) and the ΔF/F. Gray boxes show the timing of the CS. Please see the legend of Fig. 2a and Fig. 4e for explanation of the graphs.

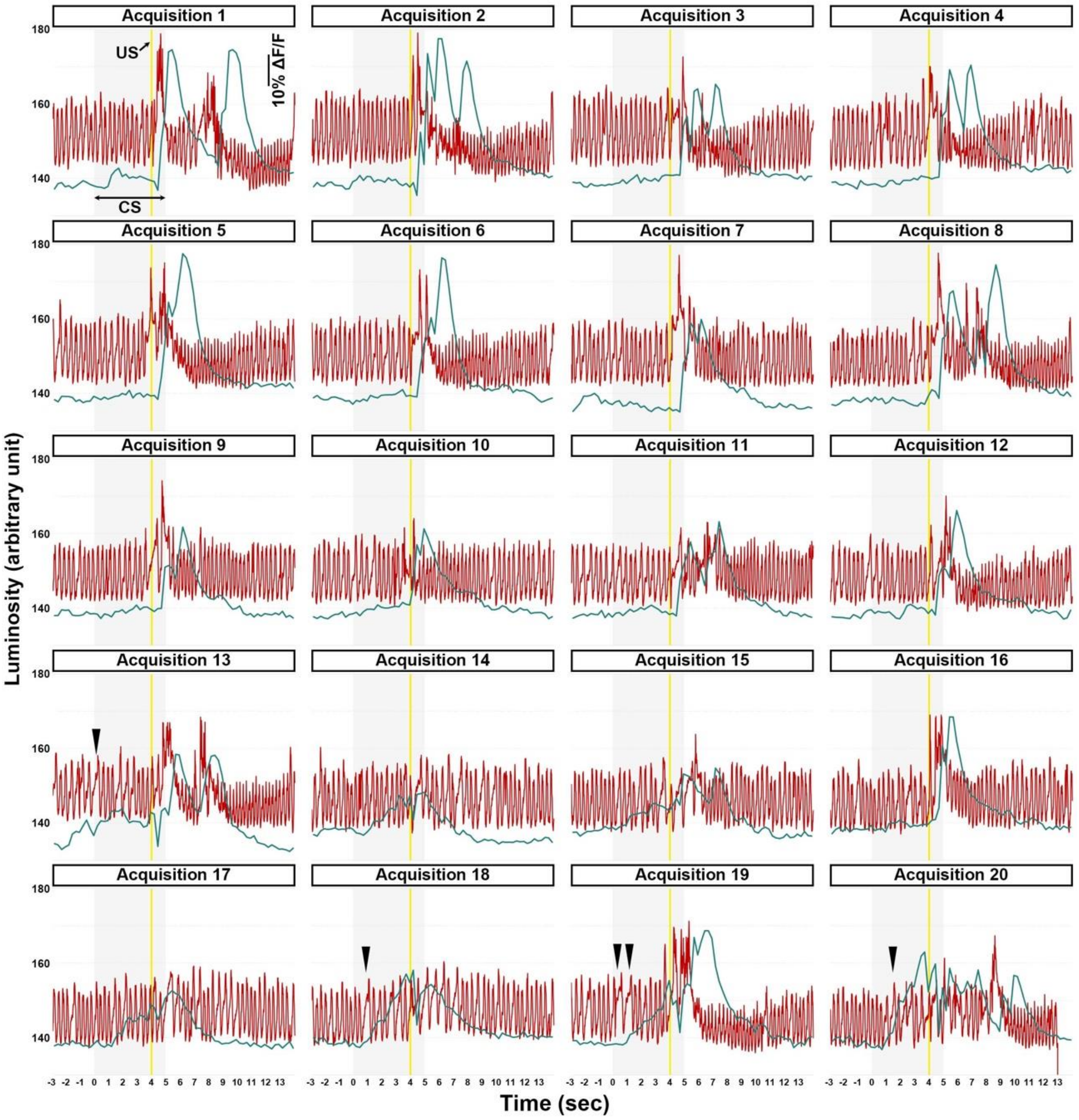

Figure S6 Simultaneous monitoring of the neuronal activity and the HB pattern of a learner larva in the acquisition session.

The HB pattern (red lines) and the ΔF/F activity of a type I conditioning-associated neuron (green line) in the learner in the acquisition session are shown. The x-axis shows the time (sec). The y-axis shows the HBs as monitored by luminosity (arbitrary unit) and the ΔF/F. Gray boxes show the timing of the CS; yellow lines show the timing of the US. The conditioned bradycardia responses are indicated by arrowheads. Note that the conditioned bradycardia response was first detected in the 13th trial when the ΔF/F increase was apparently observed.

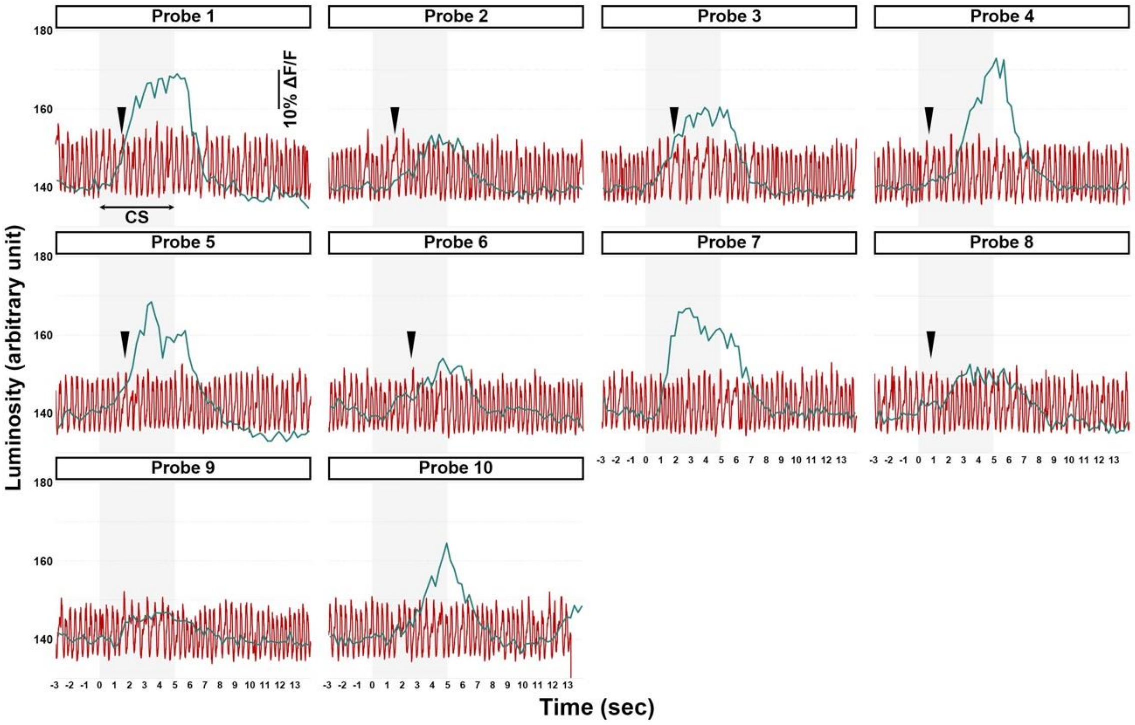

Figure S7 Simultaneous monitoring of the neuronal activity and the HB pattern of a learner larva in the probe session.

The HB pattern (red line) and the ΔF/F activity of a type I conditioning-associated neuron (green lines) in the learner in the probe session are shown. The x-axis shows the time (sec). The y-axis shows the HBs as monitored by luminosity (arbitrary unit) and the ΔF/F. Gray boxes show the timing of the CS. The conditioned bradycardia responses are indicated by arrowheads.

Supplementary Videos

Video S1 Heart beats of a wild type larva in classical fear conditioning.

An about 20-dpf wild-type zebrafish larva was monitored from the ventral side with an infrared camera. Movie data from the 15th trial of the habituation session, the first trial of the acquisition session, and the 8th trial of the probe session are shown. The movie is displayed in real time. Time scale is indicated.

Video S2 Heart beats of a GC-silenced larva in classical fear conditioning.

A GC-silenced, gSA2AzGFF152B; *Tg(UAS:BoTxBLC-GFP)*, larva was monitored from the ventral side with an infrared camera. Movie data from the 8th trial of the probe session is shown. The movie is displayed in real time. Time scale is indicated.

Video S3 Ca^{2+} imaging of cerebellar neurons in classical fear conditioning.

Fluorescence image in the cerebellum of a *Tg(elavl3:GAL4-VP16); Tg(UAS:GCaMP7a)* larva were acquired. Movies from the 13th trial of the habituation session and 4th trial of the probe session are shown. Time scale is indicated.
